# Supplementary figures and images for: Functional architecture for speed tuning in primary visual cortex of carnivores
Source: bioRxiv. 2025 Nov 5:2025.11.04.686504. Preprint. [Version 1] doi: 10.1101/2025.11.04.686504 (PMC12637459; doi:10.1101/2025.11.04.686504)

# Speed Index (sorted)

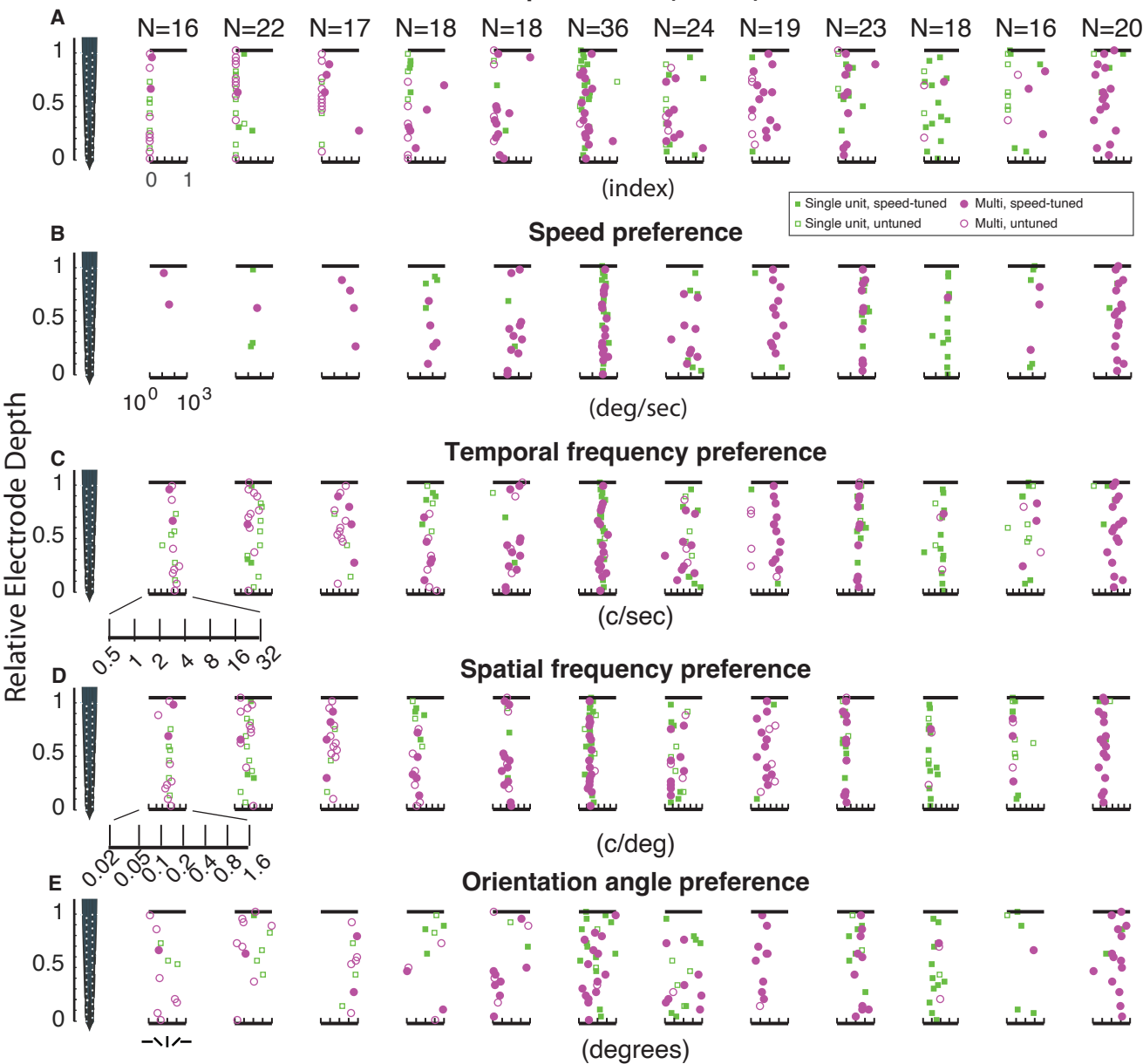

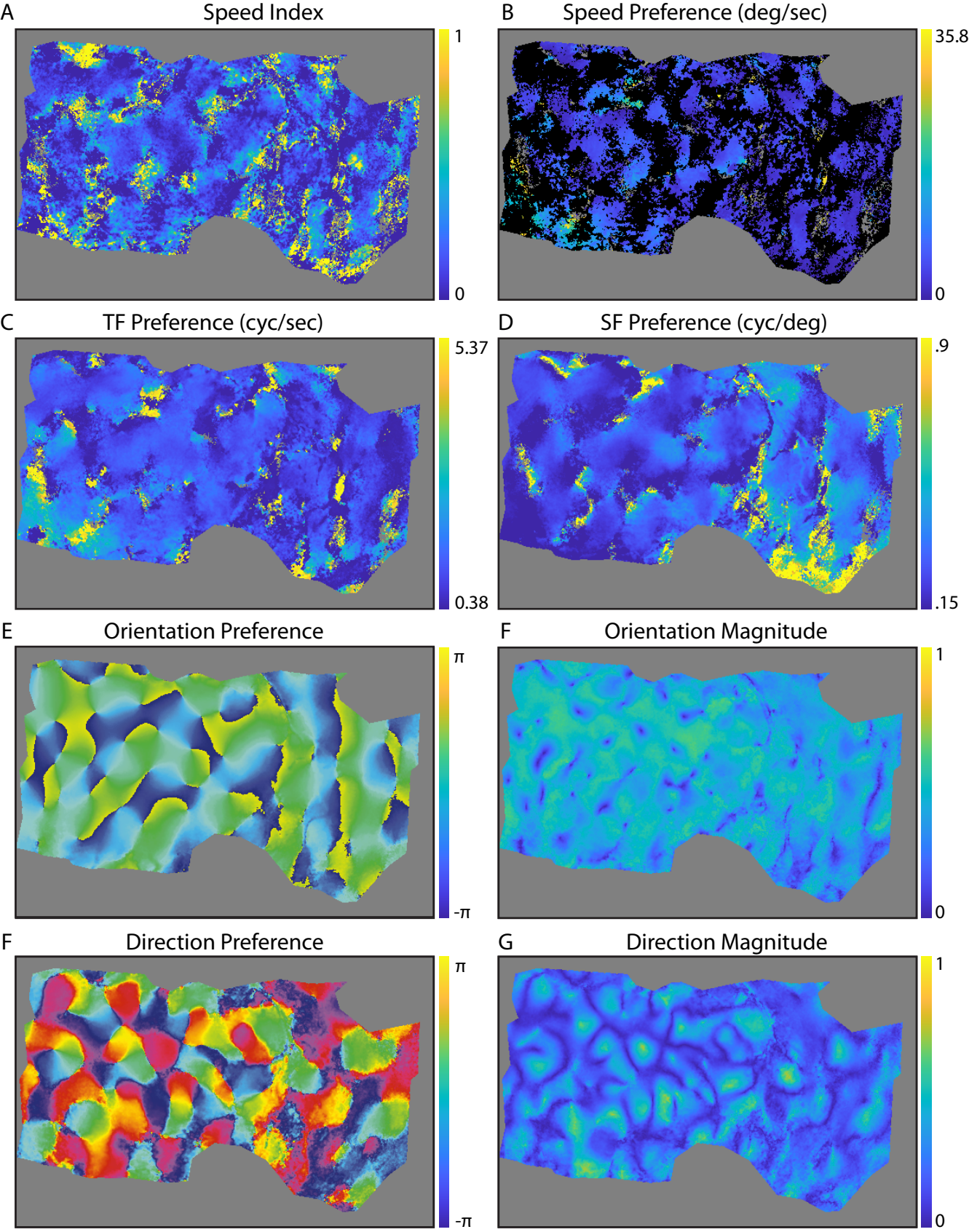

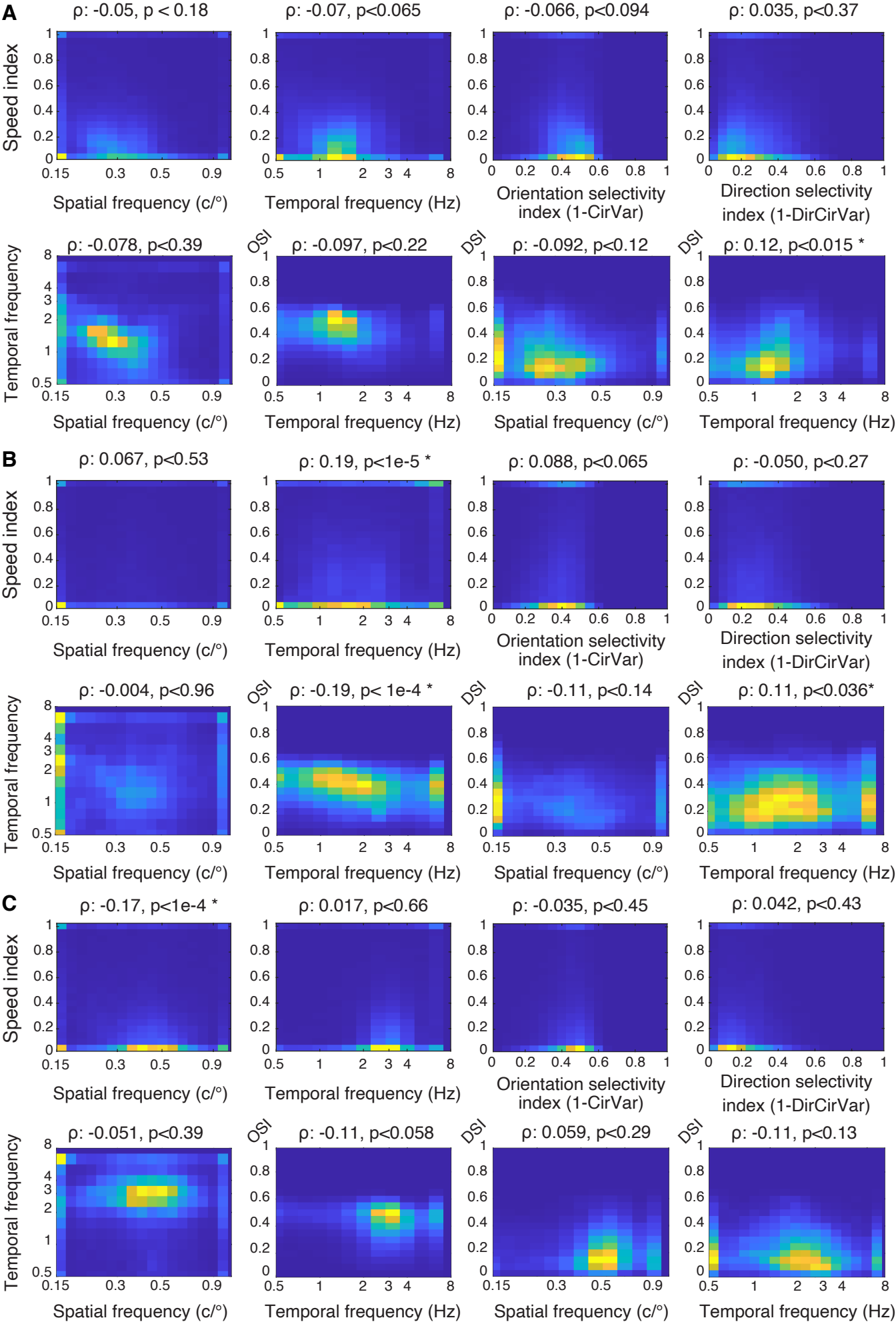

Supplement: 1 — Supplementary Figure 1. Tuning properties of cells in individual electrode penetrations. C) Temporal frequency preference. D) Spatial frequency preference. E) Orientation preference. All penetrations exhibit tuning properties that are more similar within the electrode penetration than across electrode penetrations. Supplementary Figure 2. Speed tuning hot spots in cat primary visual cortex for one subject. (A) False-color map of speed tuning index as before. (B) Speed preference for pixels that exhibit significant speed tuning by the nested F test. (C) Preferred temporal frequency from the Priebe et al. (2006) function. Temporal frequency preference values are close to 2 Hz over much of the cortex, with some small zones of high temporal frequency preference. (D) Spatial frequency preference from the Priebe et al. (2006) function. There is a gradient from low (left side) to high (right side). (E) Orientation preference angle. (F) Orientation vector magnitude; orientation index values are generally high over the cortex. (G) Direction preference map. (H) Direction magnitude map. Known regions of high direction selectivity and direction fractures (regions of low direction selectivity that form lines) are observed (Weliky et al., 1996; Ohki et al., 2005). Supplementary Figure 3. No consistent or strong correlations between speed index values and preferred spatial frequencies, preferred temporal frequencies, orientation selectivity index values, or direction selectivity index values. A) For one cat, densities (2-dimensional histograms) of speed index values and spatial frequency preferences, temporal frequency preferences, and 1-CirVar and 1-DirCirVar. In the second row, densities of relationships between non-speed parameters are shown. Spatial frequency, and temporal frequency are on log scales. Correlation coefficients (ρ) and p values calculated using shuffled maps to remove the autocorrelation of each parameter (see methods) are indicated. Colors indicate the number o [file NIHPP2025.11.04.686504v1-supplement-1.pdf]
